# Evaluation of Shear Bond Strength between Resin Composites and Conventional Glass Ionomer Cement in Class II Restorative Technique—An In Vitro Study

**DOI:** 10.3390/ma15124293

**Published:** 2022-06-17

**Authors:** Afreen Bilgrami, Afsheen Maqsood, Mohammad Khursheed Alam, Naseer Ahmed, Mohammed Mustafa, Ali Robaian Alqahtani, Abdullah Alshehri, Abdullah Ali Alqahtani, Shahad Alghannam

**Affiliations:** 1 Department of Dental Materials, Fatimah Jinnah Dental College, Karachi 74900, Pakistan; afreenagha@hotmail.com; 2 Department of Oral Pathology, Bahria University Dental College, Karachi 74400, Pakistan; afsheenmaqsood@gmail.com; 3 Department of Preventive Dentistry, College of Dentistry, Jouf University, Sakaka 72345, Saudi Arabia; 4 Prosthodontics Unit, School of Dental Sciences, Health Campus, Universiti Sains Malaysia, Kota Bharu 16150, Malaysia; 5 Department of Prosthodontics, Altamash Institute of Dental Medicine, Karachi 75500, Pakistan; 6 Department of Conservative Dental Sciences, College of Dentistry, Prince Sattam Bin Abdulaziz University, Al-Kharj 11942, Saudi Arabia; ma.mustafa@psau.edu.sa (M.M.); ali.alqahtani@psau.edu.sa (A.R.A.); am.alshehri@psau.edu.sa (A.A.); aa.alqahtani@psau.edu.sa (A.A.A.); 7 Department of Clinical and Patient Affairs at Dental College, Prince Sattam Bin Abdulaziz University, Al-Kharj 11942, Saudi Arabia; s.alghannam@psau.edu.sa

**Keywords:** sandwich technique, shear bond strength, glass ionomer cement, microhybrid composites, nanocomposites

## Abstract

The success of dental restorations depends mainly on the ability to bond to other filling materials and tooth substances, in order to resist the multitude of forces acting on the bond within the oral cavity. Although the shortcomings of composite resins have been significantly reduced over the past three decades, microleakage due to shrinkage under masticatory loads is unavoidable. In order to overcome such problems, two materials laminated with matched properties can be used to achieve optimum results. The sandwich technique is an approach in which dentine is replaced by glass ionomer cement (GIC), and enamel is replaced by composite resin. In the past, numerous materials have been proposed with adequate properties to be used in this manner, but the results are conflicting in terms of bonding to the various forms of GIC, and the appearance of microcracks or gap formation during functional loading. This study aimed to evaluate the shear bond strength (SBS) and mode of failure between the following core materials: composite resins (CR) (Methacrylate Z350™, Ceram X™, and Spectrum™) with a base material of glass ionomer cement (GIC, Ketac Molar™). Eight samples were made with the help of polytetrafluoroethylene sheets (TEFLON, Wilmington, DE, USA). Each sheet consisted of holes which were 4 mm in diameter and 2 mm in thickness. The combination of materials was sandwiched. The samples were stored in distilled water and then placed in an incubator for 24 h in order to ensure complete polymerization. The samples were thermocycled for 500 cycles between 5–55 °C/ 30 s. Following thermocycling, SBS testing was performed using a universal testing machine. Additionally, scanning electron microscopy (SEM) was performed on representative samples for the bond failure analysis between GIC and the composite resins. The Ceram-X™ nanocomposite showed significantly higher bond strength than Methacrylate Z350™ or Spectrum™ (*p* = 0.002). The Methacrylate Z350™ and the Spectrum™ composite specimens demonstrated a similar SBS (*p* = 0.281). The SBS of the Ceram X™ to GIC was the highest compared to Methacrylate Z350™ and Spectrum™. Therefore Ceram X™ may produce a better bond with GIC, and may protect teeth against recurrent caries and failure of the restoration. Methacrylate Z350™ is comparable to Spectrum™ CR and can be used as an alternative. A combination of adhesive and mixed failure was observed in Methacrylate Z350™ CR and GIC, while adhesive failure was predominantly found in both Ceram X™ and Spectrum™ with GIC restorations.

## 1. Introduction

The bilayered technique is one of the recommended techniques of composite restoration used in restorative dentistry. The technique is known by various names, such as sandwich technique or double laminate technique. The idea of the sandwich technique is to apply two restorative materials in order to form a single strong and reliable restoration [1]. The rationale behind applying the technique is to make the most of the physical and esthetic properties of each material as it combines the dentin adhesion and fluoride release of glass ionomer, as well as possesses the aesthetics and polishability of resin [2].

The composite resins are modified over time to achieve the best possible restorative outcome. The unwanted properties of composite resins such as polymerization shrinkage have been minimized as a result of the introduction of bulkfill, nano resins, and micro-hybrid resins. Advancements in adhesive dentistry have helped tremendously in overcoming the drawbacks of composite resins over the years. Furthermore, the clinical techniques for manipulating dental composites chairside have been revolutionized over the past 30 years. However, the slightest changes or shrinkage in composite cores due to thermal changes and masticatory forces is unavoidable, and these changes can lead to microleakage and recurrent caries [3,4].

In order to overcome these problems, the benefits of glass ionomer cement (GIC) and the strength of composites can be combined together to produce optimal results [5,6,7]. Although GIC has poor physical and mechanical properties, such as decreased wear resistance, toughness, strength, and brittleness, one cannot ignore the benefits of GIC, i.e., biocompatibility with dental pulp, and resistance to dental caries [8,9].

Ceram X nano-resin composite is a methacrylate-modified silicon dioxide that contains light-cured resin restorative material. It is a mixture of hybrid and modified ceramic nano-glass filler particles. This composite possesses great mechanical and optical properties, and also exhibits adequate gloss retention. Its wear resistance is almost equivalent to other composites [10,11]. Methacrylate Z350 XT nanocomposite is another material also known for its wear resistance, polishability, and retention of its gloss, in both anterior and posterior restorations. Thus, both of these materials can be used in a sandwich technique [12].

Polymerization shrinkage and resultant microleakage persist as problems with the bilayered restorative technique. Shrinkage is due to a problem in the bonding between restorative material and the substrate (GIC and CR) [6]. Microleakage and bond strength between tooth structure and filling materials have been investigated by different researchers, and it has been emphasized that the bond strength between the base and bulk filling materials is of paramount importance in restorative dentistry [4,7,9]. Therefore, it is also important to check manufacturers’ claims of high bond strength between restorative materials. The current study used the sandwich technique to evaluate the bond strength of both aforementioned restorative materials. Since various factors like polymerization shrinkage, microleakage, and bond failure are related to the dislodgement of filling materials from cavities, investigation needs to be carried out in order to determine the factors responsible for bond failure between bulk restoration and lining materials. This study aimed to evaluate the shear bond strength (SBS) and mode of failure between the following: conventional glass ionomer cement, GIC (Ketac Molar™, 3M ESPE, St. Paul, MN, USA) and bulk materials, and nanocomposites Ceram X™ (DENTSPLY SIRONA, Charlotte, NC, USA) and Z350 (3M ESPE, St. Paul, MN, USA) compared with microhybrid composite Spectrum™, DENTSPLY SIRONA, Charlotte, NC, USA). The hypothesis of this study was that there is a difference between the shear bond strength and mode of failure of composite resin versus conventional glass ionomer cement.

## 2. Materials and Methods

### 2.1. Study Design and Setting

The current study was an experimental trial. Samples were made at the Dr. Ishrat-ul-Ebad Khan Institute of Oral Health Sciences, Pakistan,. The samples were sent to the dental materials laboratory in a temperature-controlled container, as per manufacturer’s instructions, in order to test the SBS on a Universal Testing Machine (Zwicki Z5.0, Zwick Roell Group, Ulm, Germany) with particular specifications (200 N loadcell HP, 3–4 mm diameter clamping device, *x*-*y* axis moveable table; Alu t-slot plate). The pre-load was 0.1 N at a test speed of 1 mm/min.

### 2.2. Samples Preparation

The samples were prepared by an expert operator (A.F.) in a polytetrafluoroethylene (TEFLON, Wilmington, DE, USA) mold. The mold consisted of two parts; each part was 2 mm thick, with a diameter of 4 mm. Teflon sheets were fixed over a metallic base in order to avoid any error. GIC was filled in the first sheet and then left for the initial setting. During setting time, the top and bottom surfaces of the material were covered with cellulose acetate strips, and small glass slides were used in the microscopes. A 37% phosphoric acid gel was applied for 20 s, then the surface of GIC was rinsed and dried, and then RC was filled in the second part of mold placed over the first sheet, overlapping the holes of the first sheet. The RC was covered with a cellulose strip on top and cured for 40 s. The prepared samples were carefully taken out of the mold and inspected for any errors. Two samples from group A were removed due to inaccurate dimensions, and one sample each from groups B and C was removed as a result of porosity. A total of eight final inspected samples were included in each group. The samples were then placed in dark brown labeled bottles filled with distilled water. The bottles were placed in an incubator (Jiangsu XCH Biomedical Technology Co., Ltd., Taizhou, China) at 37 °C for 24 h, in order to allow for the complete polymerization of RC and setting reaction of GIC. The samples were then placed in a thermal cycler P × 2 (Thermo Electron Corporation, Waltham, MA, USA) for 500 cycles between 5–55 °C/30 s. The bottles were stored in a sealed, temperature-controlled carrier before being transported to the universal testing machine for shear bond strength evaluation.

### 2.3. Materials Included in the Study

The materials used in this study were:

- Methacrylate Z350™ (3M ESPE);
- Ceram X™ (DENTSPLY, SIRONA);
- GIC (Ketac Molar™, 3M ESPE);
- Microhybrid composite (Spectrum™ (DENSTPLY, SIRONA);
- Phosphoric acid gel (3M ESPE & DENTSPLY).

The composition, types, and brands of materials used in this study are described in Table 1.

### 2.4. Sample Size Calculation and Grouping

The sample size was calculated using Open-epi software, considering the lowest mean shear bond strength values of 9.98 ± 3.15 [13], with a confidence interval of 95% and margin of error 5%. The power of test was 80%. The estimated sample size for specimens in this study was 24, which was divided into three groups:

Group A: 08 samples (GIC + composite A, Z350™ (3M ESPE, St. Paul, MN, USA).
Group B: 08 samples (GIC + composite B, Ceram X™ (DENTSPLY SIRONA, Charlotte, NC, USA).
Control group C: 08 samples (GIC + Microhybrid composite C, Spectrum (DENTSPLY SIRONA, Charlotte, NC, USA).

### 2.5. Sampling Technique and Criteria

The purposive randomized sampling method was used in this study. The samples included were made up of Methacrylate Z350™ (3M ESPE, St. Paul, MN, USA), Spectrum^™^ (DENTSPLY SIRONA, Charlotte, NC, USA), and Ceram X™ (DENTSPLY SIRONA, Charlotte, NC, USA) with GIC (Ketac Molar™, 3M ESPE, St. Paul, MN, USA). The lamination technique (GIC + RC), was used according to the manufacturer’s recommendations. A second-generation LED light-curing unit (440 nm to 500 nm, Translux Power Blue™, Heraus Kulzer, Germany) caliberated at 40 s was used for curing the specimens.

The damaged samples, such as those having cracks or air bubbles, were not included. The samples which were not completely cured were also excluded.

### 2.6. In Vitro Testing Machine Specs and Configuration

- Universal Testing Machine (Zwicki Z5.0, Zwick Roell Group, Ulm, Germany);
- 200-Newton load cell HP;
- 3–4-millimeter diameter clamping device;
- *x*-*y* axis moveable table;
- Alu t-slot plate.

The samples were tested for shear bond strength, adopting the “ISO 29022:2013” standard. The (Zwicki Z5.0, Zwick Roell Group, Ulm, Germany) 200-Newton load cell HP, 3~4-millimeter diameter clamping device was used with an *x*-*y* axis moveable table and Alu t-slot plate. The preload used for testing was 0.1 N, and the test speed was 1 mm/min.

### 2.7. In Vitro Testing Process Pictorial Description

**Figure 1:**
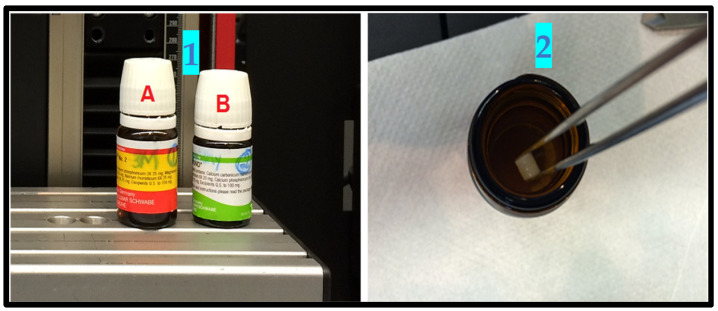
1(A): Methacrylate Z350 1(B): Ceram X (**left side**). 2: The samples were stored in distilled water in a colored brown bottles (**right side**).

Step 1: The prepared samples were preserved in distilled water, for the first and second tests for Material 1A, Methacrylate Z350 (3M ESPE, St. Paul, MN, USA) and Material 1B Ceram X™ (DENTSPLY SIRONA, Charlotte, NC, USA), shown on the left side of Figure 1. The control Spectrum™ CR (DENTSPLY SIRONA, Charlotte, NC, USA) was kept in a similar colored bottle.

**Figure 2:**
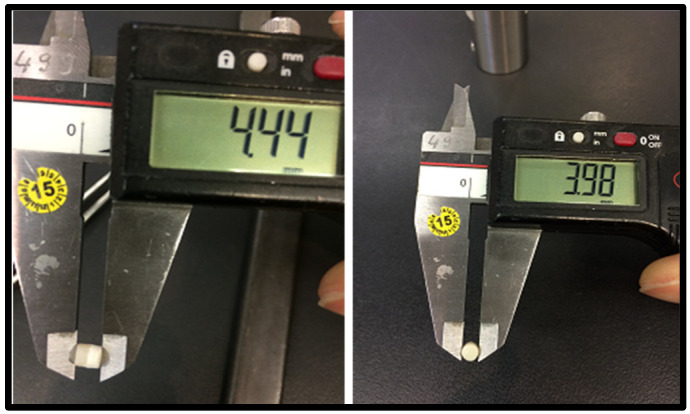
Calibration of the prepared restorative material samples.

Step 2: The samples were picked out of the distilled water with dental tweezers, in order to measure the diameter and height of the samples, Figure 2.

**Figure 3:**
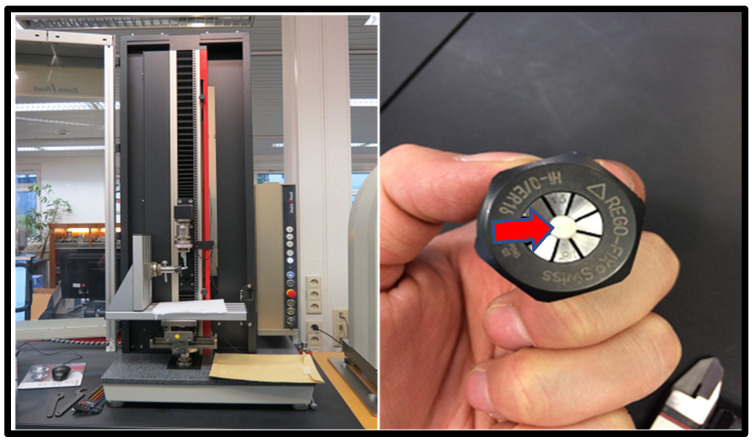
Universal Testing Machine (**left**), spiral grip device with a sample in the center ((**right**), indicated by red arrow).

Step 3: The UTM with a special clamping device to simulate 90-degree shear testing was used for SBS. A 3–4-millimeter (diameter) spiral holding device to clamp 2 mm of each specimen on one side was utilized for gripping, Figure 3.

**Figure 4:**
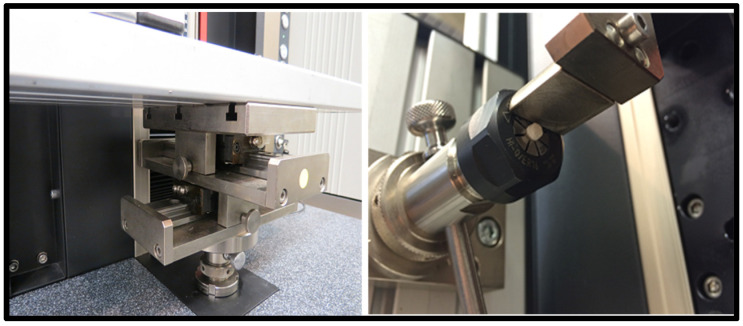
Sample fixed in the UTM before SBT; distant view (**left**), closer view (**right**).

Step 4: The *x*-*y* axis moveable table was connected with the Alu testing plate which can adjust samples to exactly align in the middle of the upper indenter, Figure 4.

**Figure 5:**
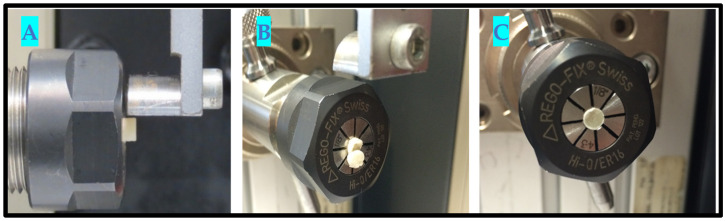
(**A**) Intact sample, (**B**) sample after force was applied, (**C**) junction of one restorative after testing.

Step 5: The specimens with 0.1-Newton preloading at test speed 1 mm/min with a 40% of maximum force was applied. The distance between the clamp device and the upper indenter was 0.7–1 mm (depending on the height of samples), Figure 5.

**Figure 6:**
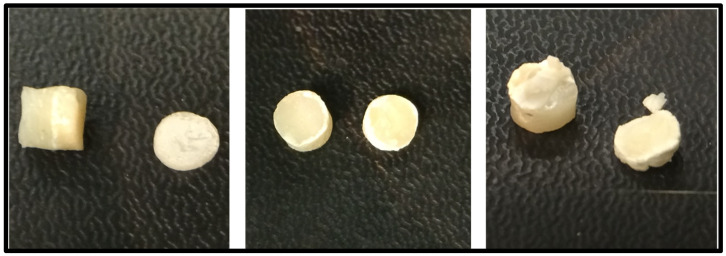
The failure modes after testing: adhesive failure (**left**), cohesive failure (**middle**), and mixed failure (**right**).

Step 6: The faiure modes of bond between CR and GIC is demonstrated in Figure 6.

Finally, the sample was stored for future reference in a sealed lock plastic bags.

### 2.8. Scanning Electron Microscopy (SEM) Analysis

Six representative samples of each group were observed under a microscope using a magnification of between 100× and 10,000×, and mounted on scanning electron microscopy (SEM) studs. The specimens were kept in distilled water for a week, air dried for two hours, mounted on SEM stubs so that the relevant area could be seen, sputter coated with 10 nm of gold in a Polaron E5100 SEM coating unit (Polaron Equipment Ltd., Hertfordshire, UK), and examined in a Hitachi-S-2500 SEM (Hitachi Ltd., Tokyo, Japan) at an operating voltage of 10 kV. Scanning electron micrographs were used to analyze the fracture interface between GIC and composites for bond failure, after shear bond strength testing.

### 2.9. Statistical Analysis

The data were analyzed using the Statistical Package for the Social Sciences (SPSS) statistical software, version 25 (SPSS Inc., Chicago, IL, USA). The Kolmogorov–Smirnov test was used to evaluate the normality of the data distribution. No significant difference, i.e., K.S value ˃ 0.05, was found in the distribution of study variables (SBS values), which warranted parametric test application in further analysis of the data. Initially, the descriptive analysis was carried out for mean and standard deviations. The mean bond strengths of the groups were compared using one-way analysis of variance (ANOVA), and Tukey’s post hoc test was used for inter-group comparison (*p* ≤ 0.05).

## 3. Results

The analysis of Z350 is presented in Table 2. The study results showed adhesive bond failure, cohesive bond failure, and mixed-mode bond failure for the materials tested. The Methacrylate Z350™ material had a total of eight observations. The first pretest observation provided an SBS (kPa) of 292. Observations 2, 4, and 7 showed bond strengths (kPa) of 108, 681, and 2367, respectively, and all these observations showed adhesive failure. Moreover, observations 3, 6, and 8 were mixed failures showing shear bond strengths (kPa) of 1223, 3404, and 1549, respectively. Only the fifth observation with SBS (kPa) of 2255 resulted in cohesive failure. Furthermore, the mean (×) value of SBS was 1485 kPa, whereas the standard deviation (S.D.) was found to be 1140 kPa. The analysis showed that the average bond strength required to contract the resin composite material and resin restoration for Methacrylate Z350™ (3M) was 1485 kPa, and this deviated up to 1140 kPa, causing the range of the Methacrylate Z350™ (3M) to be between 2625 kPa and 345 kPa. The mean value for group A was 1484.96 ± 1139.64 kPa, or 1.484 ± 1.139 MPa.

The results of the second material, i.e., Ceram X™, are shown in Table 3. The shear bond strength of all the observations and failure modes on an individual basis were as follows: the analysis of Methacrylate Z350™ and the Ceram X™ had also a total of eight observations in the analysis; initially, seven observations had adhesive failure, whereas only the eighth observation had cohesive failure. Additionally, the mean of bond strength for Ceram X™ was found to be 6063 kPa with a standard deviation of 2547 kPA, as shown in the above table. The highest shear bond strength was associated with the fourth observation, i.e., 10,779 kPa, and the lowest bond strength was associated with the sixth observation, i.e., 2543 kPa; both the above-stated observations were failed in adhesive mode. As for Ceram X™, the study results yielded that the average requirement for the contraction of resin composite material and GIC restoration is a bond strength of 6063 kPa, with a possible deviation between 8610 and 3516 kPa in bond strength. The mean value for group B was 6062.76 ± 2547.39 kPa, or 6.062 ± 2.547 MPa.

The results of the third material, i.e., Spectrum™, are shown in Table 4. The shear bond strength of all the observations and failure modes on an individual basis were as follows: there were a total of eight observations in the analysis; the first observation showed that there was cohesive failure, and the remaining seven observations were adhesive bond failures between RC Spectrum™ and conventional GIC. Furthermore, the mean of bond strength for this material was 1974.363 kPa with a standard deviation of 21.76 kPa. The mean shear bond strength in MPa was 1.974 ± 0.841. The highest shear bond strength was associated with the third observation, i.e., 2402 kPa, and the lowest bond strength was associated with the eighth observation, i.e., 1590 kPa. Likewise in group B material, both the above-stated observations were failed in adhesive mode. As for Ceram X™, the study results yielded that the average requirement for the contraction of resin composite material and GIC restoration is a bond strength of 1975 kPa.

The highest SBS values (6.062 ± 2.547) were recorded for Ceram X™. Although the Ceram X™ composite showed significantly higher bond strength to Methacrylate Z350^®^ and conventional composite Spectrum^®^ (*p* < 0.001), the Methacrylate Z350™ + GIC showed similar bond strength performance with conventional composite Spectrum™, as presented in Table 5. The post hoc test indicated that the Ceram-X nanocomposite showed significantly higher bond strength to Methacrylate Z350™ and Spectrum™ (*p* = 0.002). The Z350 composite and the Spectrum specimen, however, demonstrated similar SBS (*p* = 0.281).

### Scanning Electron Microscopy Images

**Figure 7:**
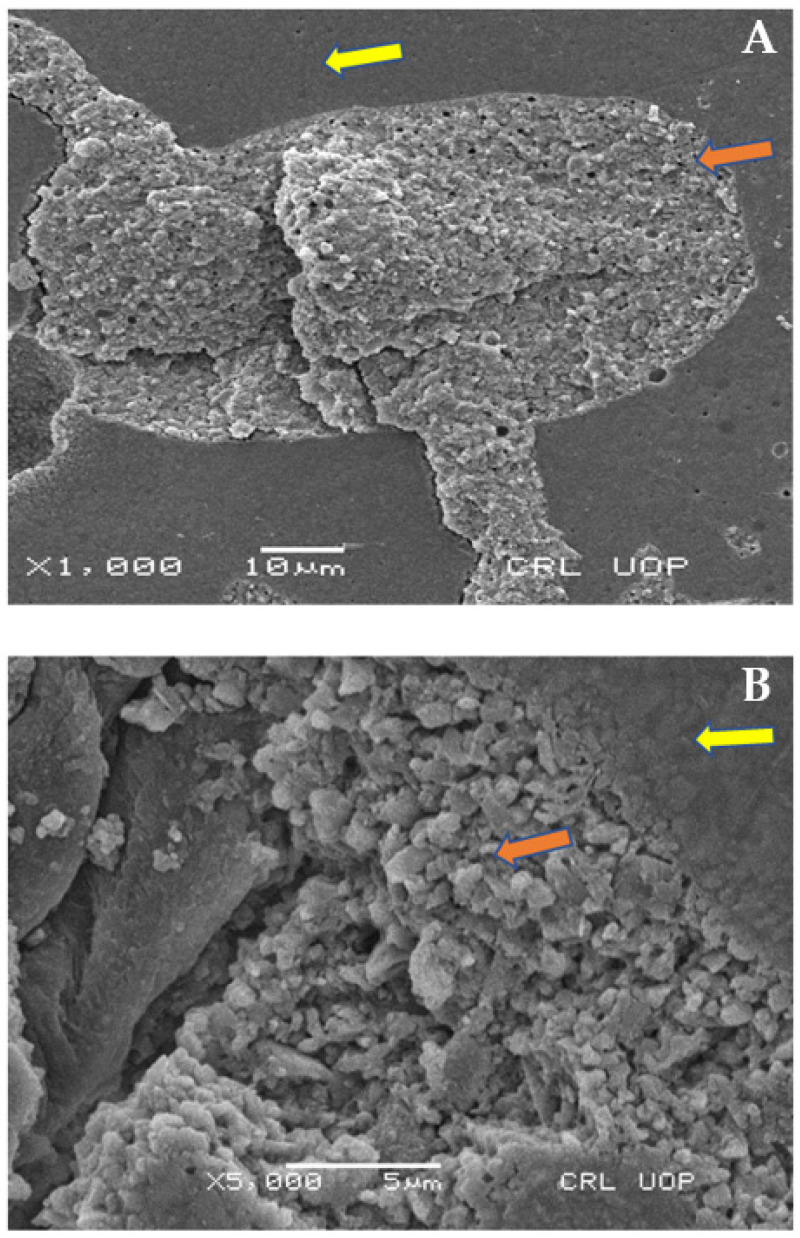
Scanning electron microscopy images showing failure modes of GIC + Z350 (Group A), mixed failure under scanning electron microscope. (**A**) Yellow and orange arrow indicates mixed failure region in low magnification 1000×. (**B**) The magnified cohesive and adhesive failure regions at 5000×.

**Figure 8:**
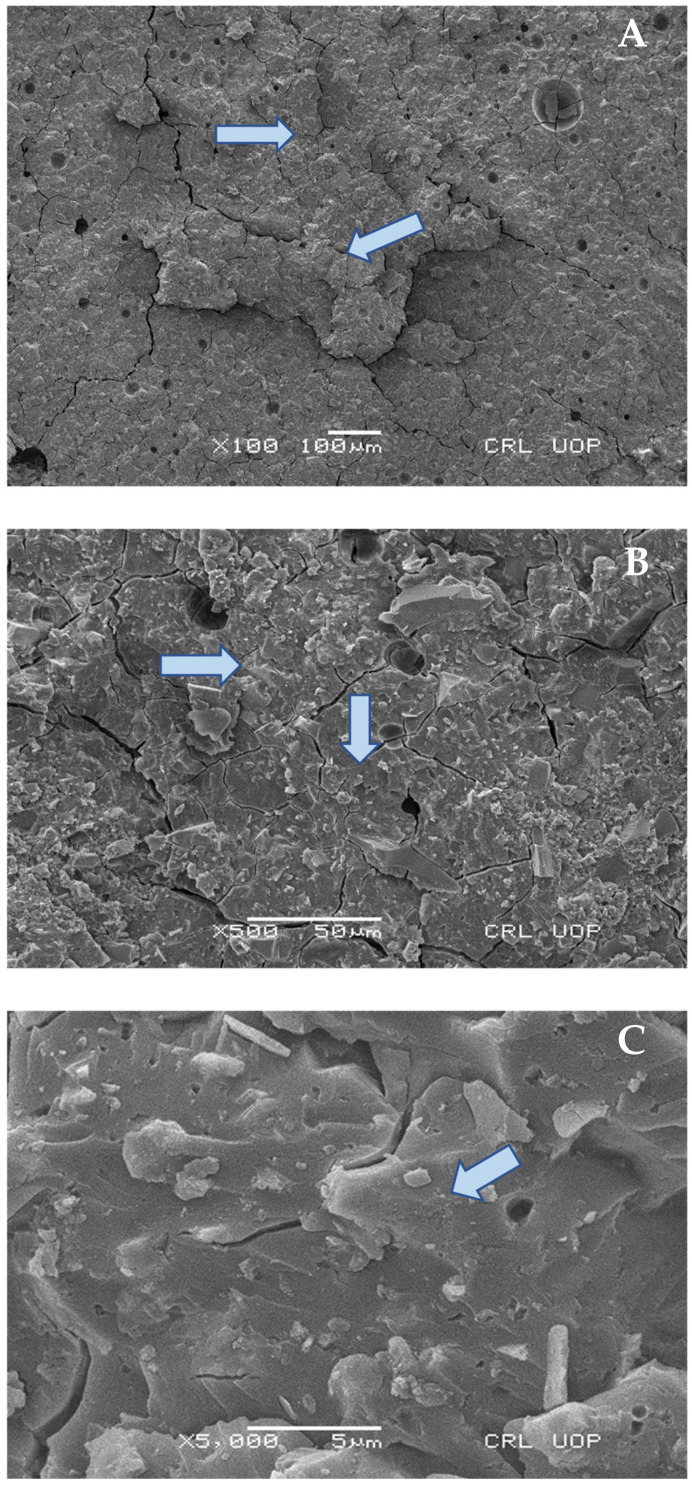
Failure mode of GIC + Ceram X (Group B), cohesive failure under scanning electron microscope. Blue arrows indicate magnified cohesive failure regions, (**A**) 100×, (**B**) 500×, and (**C**) at 5000× magnifcation.

**Figure 9:**
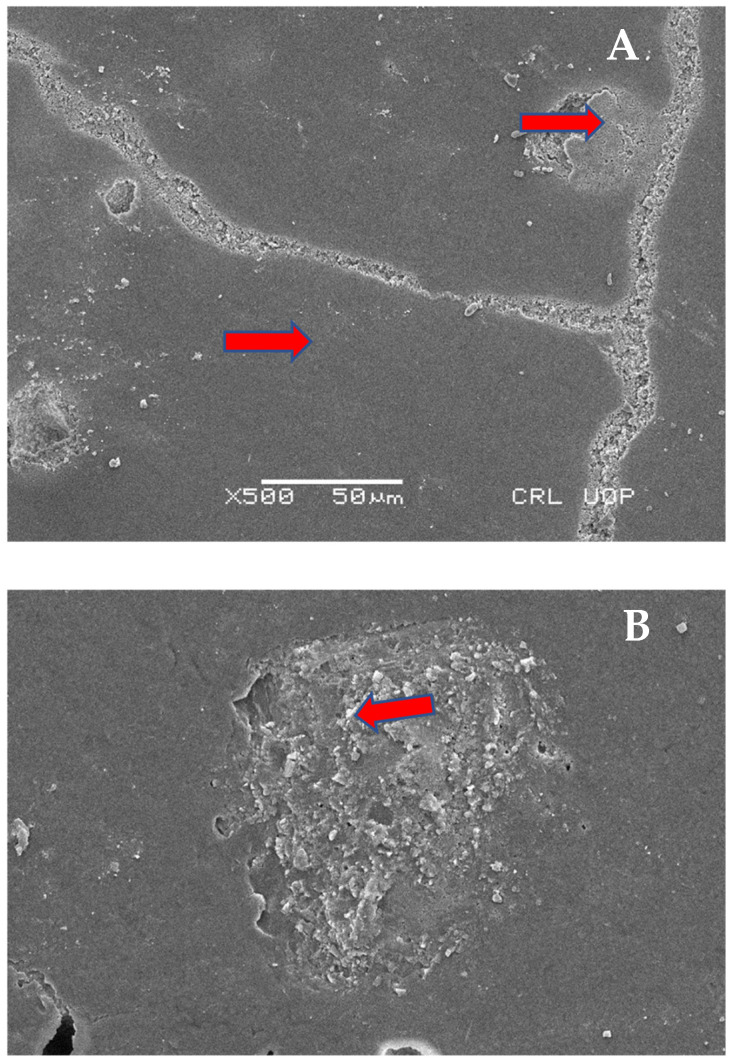
Failure mode of GIC + Spectrum (Group C), adhesive failure under scanning electron microscope. Red arrows indicate adhesive failure regions, (**A**) at 500×, and (**B**) at 1000×.

SEM analysis was performed after shear bond strength testing. Figure 7 shows mixed modes of failure failure at 1000× and 5000× magnification under scaning electron microscope, with a field area ranging from 5 to 10 µm (Group A). Figure 8 dpicts Group B analysis at 100×, 500×, and 5000× (cohesive failure mode of failure). Moreover Figure 9 shows Group C analysis at 500× and 1000×, with field areas ranging from 10 to 50 µm (adhesive failure).

## 4. Discussion

The best and most easily performable method of evaluating bond strength is by using SBS testing. This is usually done to assess the bonding efficacy of filling materials to dentin [14]. The present study was performed to evaluate SBS of three different restorative materials applied with a lamination technique. The success of the lamination technique depends upon the bonding of GIC with the composite resins. The results of the present study identified cohesive, adhesive, and mixed failures in two of the restorative materials tested. The optimal SBS was found in Ceram X™ composite resins.

Along with other advantages of GIC, its low cost and less sensitive restoration technique make it worth considering for restorative procedures. It does bond with tooth substance, but it has deficient chemical bonding with other restorative materials as a result of the dissimilar nature of setting reactions of GIC and RC. Secondly, the GIC also has an insufficient cohesive strength [12]. The reported value of SBS in conventional GIC is 3.81 MPa [9]. A reduced SBS value of conventional GIC is reported, as a result of its vulnerability to moisture [14]. The pretreatment of the tooth and GIC surface in lamination technique with acid etching carries the protocol of washing and drying the etched surface; this may result in microcracks and bond failure with RC [15]. Nonetheless, the bond strength of resin-based GIC (RMGIC) with RC is better than conventional GIC due to its hydrophobic nature that enhances bonding to other materials and tooth surfaces [10,14]. Navimipour et al. stated that SBS of RMGIC can further be improved by surface treatment with phosphoric acid and laser application [16].

The literature has proven that glass ionomer cement bond with tooth substance is weaker compared to composite bond with enamel and dentine. The GIC also has diminished cohesive potential as a result of weaker intraparticle bonding. Hence, the bond between CR and GIC is stronger than adhesion of GIC with dentine [17]. In lamination technique this can cause GIC restoration failure, especially from the tooth. In order to obtain a stronger SBS, prior treatment of tooth and CR during lamination technique is recommended. The Er,Cr:YSGG has a potential to be used for conditioning of GIC prior to composite restoration. Surface etching of tooth and RMGIC with 37% phosphoric acid prior to composite resin still remains the gold standard [18].

In this study, lower SBS values were associated with the Methacrylate Z350™ and GIC combination than group A. In contrast, Sharfuddin et al. [17] in their study showed the highest SBS values in Methacrylate Z350 CR. The reported reason for those results was because of stable polymerization chain reactions due to the incorporation of nano-hydroxyapatite crystals in CR. In the Ceram X™ and Ketac Molar™ group, higher SBS values were found and adhesive failures was predominantly seen, which could be due to the thermocycling effect that might exhaust the bond. In a study by Preethy et al. [19] on SBS analysis of different composites, the SBS of composites Ceram X™ (Densplyjor) and (Filtek Z350 XT, 3M ESP) were comparable to the results of the current study.

This study used a thermocycling protocol for both specimens during the analysis of SBS. The use of the thermocycling process yields contradictory findings in different studies. Some studies stated that it enhances the bonding capacity of restorative materials, whereas others state that it weakens the bond or has no effect on the material’s bonding ability [20,21]. The reason for such contradictory findings could originate from the different methodologies of various studies reported. The current study has the limitation of not testing the SBS of the samples before thermocycling; thus, as a consequence, the authors are unable to comment on the exact effect.

A usual protocol of lamination technique is the application of adhesive systems, either as two-step or three-step processes; etch, primer and bond, or self-etch have been reported to achieve suitable bonding to bear the masticatory forces [22]. It has been reported that prolonged acid etching weakens the topography of conventional GIC due to the effect of an acidic pH; a strong acid can cause the accumulation of salt crumps and decrease the strength of GIC. Furthermore, post-etch drying of the GIC surface may further weaken it [23]. This study incorporated acid etch protocol in order to create surface energy for CR to bond, due to its requirement of resin tags infiltration. In order to produce a distinctive outcome, in the current study and to experiment with, intentionally no adhesive system had been used. We wanted to identify the strength of the bond between known materials with distinctive properties, despite knowing the usual protocol of GIC and RC, which is widely used by clinicians. It has been reported in the scientific literature that acid etching and rinsing enhances the bonding capacity of different GIC forms, following variable methodologies [24]. Sharafeddin et al. stated that mild self-etched adhesive results in higher SBS than intermediate and strong adhesive systems [25]. However, the ideology of this research was to evaluate the bond strength of GIC, which bonds chemically with the tooth structure; we wanted to evaluate and take advantage of its chemical bonding nature, hence an additional bonding agent was not applied in this study.

There are other materials available compared to Ketac molar™ GIC with adequate strength and teeth bonding. This study focused on the use of conventional GIC; its upgraded forms like Equia Fill system were avoided due to their coated nature. In the Equia Fill system, the cement particles are coated to improve their mechanical properties. The conventional GIC has the additional advantage of reducing fluoride requirements more than coated variants, which is required to reduce caries risk [26,27,28]. The Ketac Molar™ has a wide range of use in procedures such as atraumatic restorative techniques (ART), Class II closed sandwich technique, and deciduous tooth restorations [29]. In Third World countries, its use is frequent due to its low cost, ease of availability, and handling; hence, due to the wider scope of Ketac molar in these regions, this study focused on it.

In order to avoid dislodgement, the restoration should have sufficient strength to bear masticatory forces without cracking and microleakage. All three types of bonding failure modes were observed in this in vitro study. The adhesive failure was associated mostly with Group B, suggesting that there was enhanced fracture resistance in Ceram X™ within the material; meanwhile, a combination of failures was seen in group A (Methacrylate Z350), indicating that it is a weaker material in terms of longevity since such failures would reduce the life of restorations from leakage and weakened bonds.

This study had some limitations. Firstly, a small number of specimens were incorporated for testing; in order to gain a better idea of variations in the observed data, a large sample size would have provided a sounder scientific outcome. The study may be underpowered in terms of detecting the desired difference, as a result of the smaller sample size used. Therefore, the outcomes of the study should be interpreted cautiously. Furthermore, the design of the study could only explore the inherent properties of RC materials used in the study; for a long-term success evaluation, a clinical trial with a follow-up of the patients is required. Lastly, the application of adhesives was not incorporated in the samples; the degree of shear bond strength could have been compared amongst the materials if adhesives were applied. Considering this study as a baseline, it is recommended to carry out further comparative studies in order to explore the adhesive and no adhesive application effects on the shear bond strength of layered restorations.

This study is useful because it provides a snapshot of the relation of the materials in a specific way. The results of the SBS test do not provide a reflection of the material itself, yet they indicate the behavior of the bonding between core and base material in various configurations. Altering the test materials can lead to different outcomes, and as this is an in vitro study, the results would not necessarily be the same as in clinical practice. Therefore, continuation in the form of randomized controlled clinical trials (RCTs) is required to formulate a clinically relevant conclusion. Future studies should evaluate the effects of various generations of bonding agents on the bond strength of composite resins to different types of GIC.

## 5. Conclusions

The SBS of the Ceram X™ to GIC was the highest compared to the Methacrylate Z350™ and Spectrum™ alternatives. Therefore, Ceram X™ may produce a better bond with GIC and may protect teeth against recurrent caries and failure of the restoration. Methacrylate Z350™ is comparable to Spectrum™ CR and can be used as an alternative bonding agent. A combination of adhesive and mixed failure was observed in Methacrylate Z350™ CR and GIC, while adhesive failure was predominantly found in both Ceram X™ and Spectrum™ with GIC restorations.

## Figures and Tables

**Table 1:** Composition of the materials included in the study.

Material	Type	Liquid	Powder	Brand
Ketac Molar™ Easy mix	glass ionomer	polialquenoic acid/tartaric acid/water	aluminum-calcium-lanthanum fluorosilicate glass copolymer (5% acrylic acid and maleic acid	3M ESPE (St. Paul, MN, USA)
Nanocomposite Ceram X™	polymethylmethacrylate-based resin composite	not supplied	nanoparticles and nanofillers as used in Prime & Bond NT combined with conventional glass fillers of 1µm	DENTSPLY SIRONA (Charlotte, NC, USA)
Nanocomposite Methacrylate Z350	polymethylmethacrylate-based resin composite	not supplied	Bis-GMA, UDMA, TEGDMA, and Bis-EMA resins, PEGDMA, Zirconia/Silica cluster filler	3M ESPE (St. Paul, MN, USA)
Microhybrid composite Spectrum™	polymethylmethacrylate-based resin composite	not supplied	TEGDMA ethyl4(dimethylamino)benzoate butylated hydroxytoluene (BHT) UV stabilizer. barium-aluminium-borosilicate glass (mean particle size < 1 µm)	DENTSPLY SIRONA (Charlotte, NC, USA)

**Table 2:** The distribution of shear bond strength and failure modes for Methacrylate Z350™ (3M ESPE).

Material A No	F_max_ N	Shear Bond Strength kPa	dL at F_max_ mm	d_o_ mm	S_o_ mm^2^	Failure Mode
1.1	3.639	292.49	0.007	3.98	12.44	pretest
1.2	1.327	108.28	0.030	3.95	12.25	adhesive
1.3	15.14	1222.81	0.360	3.97	12.38	mixed failure
1.4	8.429	680.97	0.200	3.97	12.38	adhesive
1.5	25.57	2254.94	0.162	3.80	11.34	cohesive
1.6	42.35	3404.32	0.105	3.98	12.44	mixed failure
1.7	27.13	2367.17	0.127	3.82	11.46	adhesive
1.8	19.17	1548.74	0.177	3.97	12.38	mixed failure

**Table 3:** The distribution of shear bond strength and failure modes of Ceram X™ (DENTSPLY SIRONA).

Material B No	F_max_ N	Shear Bond Strength kPa	dL at F_max_ mm	d_o_ mm	S_o_ mm^2^	Failure Mode
2.1	59.53	5009.21	0.181	3.97	11.88	adhesive
2.2	88.09	7450.54	0.462	3.88	11.82	adhesive
2.3	71.03	5680.66	0.380	3.99	12.50	adhesive
2.4	130.80	10,778.97	0.572	3.93	12.13	adhesive
2.5	65.33	5277.57	0.352	3.97	13.38	adhesive
2.6	30.22	2542.68	0.329	3.89	11.88	adhesive
2.7	95.84	7742.19	0.776	3.97	12.38	adhesive
2.8	49.26	4020.25	0.249	3.95	12.25	cohesive

**Table 4:** The distribution of shear bond strength and failure modes of Spectrum™ (DENTSPLY, SIRONA).

Material C No	F_max_ N	Shear Bond Strength kPa	dL at F_max_ mm	d_o_ mm	S_o_ mm	Failure Mode
3.1	16.342	1800.61	0.029	3.96	12.23	cohesive
3.2	9.786	1600.56	0.491	3.88	12.99	adhesive
3.3	22.169	2401.68	0.261	3.91	12.11	adhesive
3.4	19.794	2134.89	0.537	3.96	12.09	adhesive
3.5	27.602	1965.34	0.248	3.90	11.99	adhesive
3.6	23.661	2012.56	0.239	3.86	12.88	adhesive
3.7	20.679	2289.65	0.219	3.55	12.33	adhesive
3.8	25.810	1589.62	0.431	3.82	12.11	adhesive

**Table 5:** Shear bond strength values of resin composite and conventional glass ionomer cement.

Resin Composite	N	Mean and SD (MPa)	Group Comparison	*p*-Value
Methacrylate Z350 (3M ESPE) + GIC (Group A)	08	1.484 ± 1.139	A and B	^d^ 0.001
Ceram X (DENTSPLY) + GIC (Group B)	08	6.062 ± 2.547	A and C	^e^ 0.824
Conventional composite Spectrum (DENTSPLY) + GIC (Group C)	08	1.974 ± 0.841	B and C	^d^ 0.001

MPa, megapascal, similar superscript; lowercase letters denote statistical significance (*p* < 0.05).

## Data Availability

The data presented in this study are available on request from the corresponding author.

## Abbreviations

List of abbreviations and their description:

**S.No**	**Abbreviation**	**Full Name/Description**
1	ANOVA	One-Way Analysis of Variance
2	°C	degrees Centigrade
3	h	hour
4	ISO	International Standard Organization
5	LED	light emitting diode
6	GIC	glass ionomer cement
7	mm	millimeter
8	nm	nanometer
9	kPa	kilopascal
10	RC	resin-based omposites
11	s	seconds
12	TEFLON	polytetrafluoroethylene
13	SBS	shear bond strength
